# A Review on the Role of Pilocarpine on the Management of Xerostomia and the Importance of the Topical Administration Systems Development

**DOI:** 10.3390/ph15060762

**Published:** 2022-06-18

**Authors:** Afroditi Kapourani, Konstantinos N. Kontogiannopoulos, Panagiotis Barmpalexis

**Affiliations:** 1 Department of Pharmaceutical Technology, School of Pharmacy, Aristotle University of Thessaloniki, 54124 Thessaloniki, Greece; akapourag@pharm.auth.gr (A.K.); kontogik@pharm.auth.gr (K.N.K.); 2 Natural Products Research Centre of Excellence-AUTH (NatPro-AUTH), Center for Interdisciplinary Research and Innovation (CIRI-AUTH), 57001 Thessaloniki, Greece

**Keywords:** xerostomia, pilocarpine, topical administration

## Abstract

Xerostomia is linked to an increased risk of dental caries, oral fungal infections, and speaking/swallowing difficulties, factors that may significantly degrade patients’ life, socially- or emotionally-wise. Consequently, there is an increasing interest in developing management approaches for confronting this oral condition, at which pilocarpine, a parasympathomimetic agent, plays a vital role. Although the therapeutic effects of orally administrated pilocarpine on the salivary gland flow and the symptoms of xerostomia have been proved by numerous studies, the systemic administration of this drug is affiliated with various adverse effects. Some of the typical adverse effects include sweating, nausea, vomiting, diarrhea, rhinitis, dizziness and increased urinary frequency. In this vein, new strategies to develop novel and effective dosage forms for topical (i.e., in the oral cavity) pilocarpine administration, in order for the salivary flow to be enhanced with minimal systemic manifestations, have emerged. Therefore, the purpose of the current review is to survey the literature concerning the performance of topical pilocarpine delivery systems. According to the findings, the topical delivery of pilocarpine can be regarded as the equivalent to systemic delivery of the drug, efficacy-wise, but with improved patient tolerance and less adverse effects.

## 1. General

Xerostomia (or dry-mouth) is defined as a subjective complaint of dry mouth, which commonly exists as a consequence of reduced salivary flow (hyposalivation) [1]. Xerostomia may arise from a multitude of systemic or local etiologies [2,3]. These include polypharmacy, neck and head radiotherapy and systematic diseases, such as diabetes mellitus, sarcoidosis, systemic lupus erythematosus and the autoimmune Sjögren’s syndrome [4,5,6,7]. From a careful look at literature, one may easily conclude that the prevalence of xerostomia in the general population presents a significant divergence, reportedly affecting a percentage of adults ranging from 5.5 to 46% [8,9].

Patients suffering from dry-mouth complain about symptoms that significantly downgrade their health and their life in general, socially or emotionally. An oral dryness- associated discomfort is the earliest and the most frequently reported symptom from patients suffering from xerostomia [10,11,12]. Additionally, it has been shown that these patients present significantly increased incidence of dental caries, oral fungal infections (e.g., candidiasis), halitosis or burning mouth and periodontal disease [13,14,15]. Furthermore, dysphagia and dysgeusia have also been reported as possible clinical effects [16,17]. 

**Figure 1:**
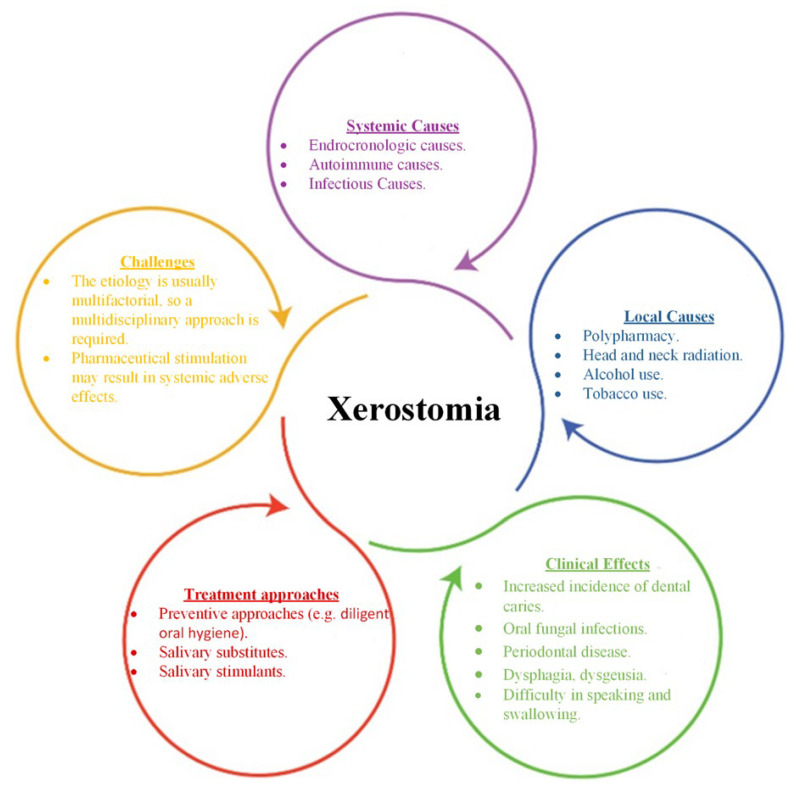
The most important aspects of xerostomia in terms of systemic and local causes, clinical effects, treatment approaches and faced challenges.

Figure 1 summarizes the most important aspects of xerostomia in terms of systemic and local causes, clinical effects, treatment approaches and faced challenges.

The etiology of the clinical manifestation determines the required therapeutic strategy, which is, in many cases, based on a multidisciplinary approach due to the multifactorial causes. The general management approach is directed at palliative treatment for the relief of symptoms and prevention of oral complications. The available treatments for xerostomia include, among others, salivary substitutes, selected in case of salivary glands’ complete damage, or salivary stimulants, chosen when salivary gland retain, at least partially, their functionality. Salivary stimulants encompass chewing gums (mechanical stimulation), malic and ascorbic acid (acid stimulation is generally avoided, despite its effectiveness, due to its potential demineralizing effect on tooth enamel [18]) and parasympathomimetic drugs (pharmaceutical stimulation) [19,20,21,22]. Various medications have been suggested as systemic sialagogues, such as pilocarpine, cevimeline and bethanechol, since they are capable of inducing the secretion of natural saliva from the undamaged part of the salivary glands through their action on muscarinic receptors. Among them, pilocarpine is the most commonly used medication and it has gained the approval of US Food and Drug Administration (FDA) for the treatment of Sjögren’s syndrome, as well as the relief of radiation-induced xerostomia’ symptoms [4]. 

## 2. Approach of the Review

In the present review, a comprehensive search was performed with the valuable aid of the electronic databases of MEDLINE/PubMed, SCOPUS, and Google scholar, which provided access to a plethora of original research articles in the English language, already published or at “in press” status in peer-reviewed literature. The terms “xerostomia”, “pilocarpine”, “systemic administration”, “salivary stimulation” and “topical administration” were used. All articles published up to March 2022 were initially reviewed with no limitations concerning the publication type.

## 3. General Information on Pilocarpine

**Figure 2:**
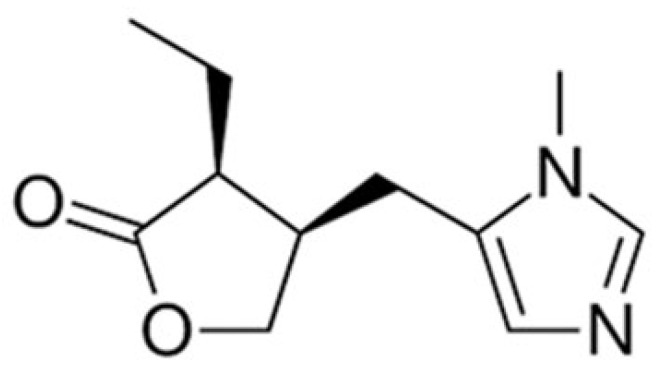
Chemical structure of pilocarpine base.

Pilocarpine is a parasympathomimetic agent that acts primarily as a non-specific muscarinic acetylcholine receptor agonist with mild beta-adrenergic activity. It is a tertiary alkaloid extracted from plants of the genus Pilocarpus and, specifically, it is found in the leaves of Pilocarpus microphyllus and Pilocarpus jaborandi [23,24,25]. Pilocarpine’s IUPAC name is (3S,4R)-3-ethyl-4-[(3-methylimidazol-4-yl) methyl]oxolan-2-one and its chemical structure is presented in Figure 2 [26]. 

### Mechanism of Action

**Figure 3:**
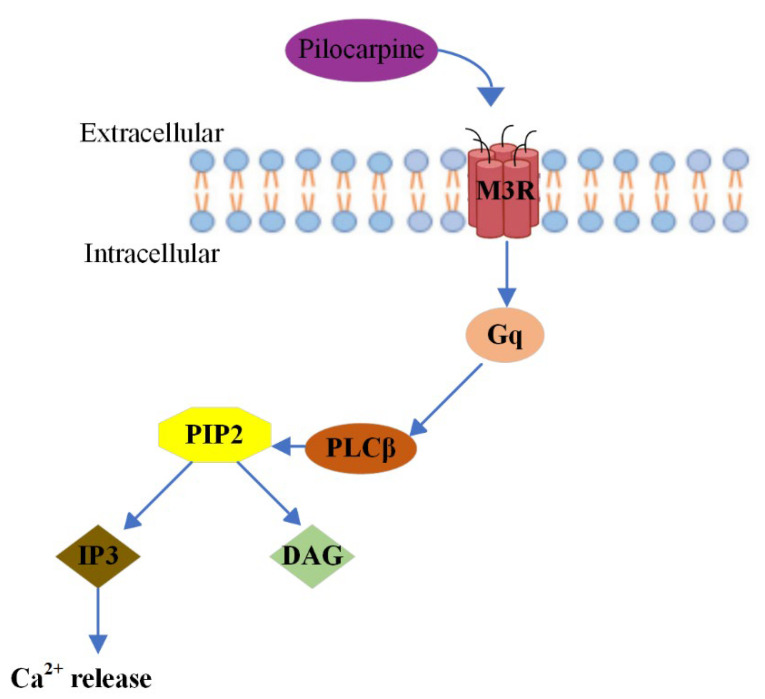
Illustration of Pilocarpine’s mechanism of action.

Pilocarpine promotes physiological salivation by binding the muscarinic acetylcholine receptor 3 (M3R) in the acinus cells of the salivary glands [27,28,29]. Generally, muscarinic acetylcholine receptors (mAChR) consist of five genetically distinct subtypes, M1–M5, and their role is pivotal in regulating a plethora of central and peripheral nervous systems’ fundamental functions [30]. Pilocarpine is capable of activating all five muscarinic receptor subtypes, but the majority of its observed therapeutic effects are mediated by M3R [31]. M3R is an excitatory receptor expressed in gastric glands, salivary glands and smooth muscle cells, such as those present in the pupillary sphincter and ciliary bodies [32]. Through stimulation of its cognate G protein, Gq, M3R activates the effector enzyme phospholipase C beta (PLCβ), which hydrolyses phospholipid PIP2, leading to the generation of the second messenger’s inositol triphosphate (IP3) and diacylglycerol (DAG) and calcium (Ca^2+^) and protein kinase [33]. Therefore, M3 cholinergic agonists result in the upregulation of calcium and ultimately smooth muscle contraction [34]. Figure 3 illustrates the Pilocarpine’s mechanism of action, as described in detail above.

## 4. Systemic Administration of Pilocarpine for the Management of Xerostomia

Until the mid-1990s, pilocarpine’s clinical application was one-dimensional and pertained essentially to the management of glaucoma. Nevertheless, during the past few decades, systemic pilocarpine was praised for its effectivity in the management of xerostomia associated with the head and neck radiation and, more recently, it has been proved to present potential effectiveness in Sjögren’s syndrome-related xerostomia [35,36,37]. 

Regarding the management of xerostomia, oral tablets represent pilocarpine’s main commercially available form. Specifically, Salagen^®^ (a pilocarpine HCl tablet) claims hitherto the monopoly in radiation-induced xerostomia treatment, being the only drug product that has gained approval, both in Europe and the USA. It is a film-coated tablet containing 5 mg of pilocarpine HCl, microcrystalline cellulose as a binder, stearic acid as a lubricant and acidifier and carnauba wax as a polishing agent. The commonly recommended dose is 2.5 to 10 mg, administered orally 3 or 4 times daily and can be adjusted, depending on patient response [38]. Central and peripheral muscarinic effects are observed within 20 min of ingestion and pilocarpine has an elimination half-life of approximately 0.76–1.3 h [39,40]. The efficacy of systemic administration of pilocarpine in increasing resting and stimulated salivary flow in diminishing subjective oral dryness and decreasing difficulties with chewing and speaking in patients with Sjögren’s syndrome and post-radiation xerostomia has been confirmed in various randomized, placebo-controlled trials, which are presented in detail in Table 1. Some of the mentioned studies are part of the most prominent clinical trials for the efficacy of the orally administrated pilocarpine against xerostomia, and, hence, in these cases, the inclusion of the clinical trial identifiers (ID) was considered important. The majority of the patients enrolled at the presented clinical studies suffered from post irradiation xerostomia. Moreover, according to the referred results, there is evidence that oral pilocarpine may provide more lasting results when administered before and continued during the radiotherapy [41].

### Adverse Effects

Although the effectiveness of the pilocarpine’s oral administration against Sjögren’s syndrome and the xerostomia-induced by radiotherapy is demonstrated by the numerous aforesaid studies, the systemic administration of this drug is affiliated with various adverse effects [63]. Typical adverse effects are sweating, nausea, vomiting, diarrhea, rhinitis, headache, chest pain, abdominal cramps, dizziness, palpitations, chills and influenza-like symptoms, increased urinary frequency and increased lacrimation [64,65,66,67,68]. Taking into consideration the pilocarpine’s pharmacological action on the exocrine glands, along with the sweat, salivary, lacrimal, pancreatic and intestinal glands, irrefutably, the afore-mentioned side effects are characterized as anticipated. Consequently, pilocarpine is not recommended to patients experiencing gastric ulcer and uncontrolled asthma, whilst the remarkable possibility of the cardiovascular effects’ appearance, following the systemic administration, is also a matter worth taking into consideration [69,70,71]. 

It has been observed that the significant systemic adverse effects caused by the ingestion of pilocarpine’s oral formulations mean that the medication has low tolerance, resulting in patient’s decreased adherence to the treatment. Sweating is the most commonly reported adverse effect and it is presented in a dose-dependent fashion [72,73]. Characteristically, Rieke et al. [49] revealed that 29% of the patients treated with 5 mg pilocarpine t.i.d. reported sweating, while in the case of the patients treated with 10 mg pilocarpine t.i.d., the respective incidence rate was 68%. In this study, considering both the safety and the efficacy of the evaluated doses, the dosage of 5 mg t.i.d. was regarded as the optimal treatment dosage, balancing clinical improvement with adverse effects. However, the same oral pilocarpine dosage (i.e., 5 mg t.i.d.) in a trial by Nakamura et al. [74] resulted in the significant prevalence of intolerable side effects for the participating patients, while the most frequently reported adverse reaction was, once again, sweating, and its rate of occurrence was 64%. Other reported side effects were nausea, rhinitis, headache, cervical pain, fatigue, dazzling and paresthesia of the tongue. 

Interestingly, according to the study conducted by Noaiseh et al. [75], at which a comparison of two different sialagogues—pilocarpine and cevimeline—took place, pilocarpine was related to higher disappointment rates among first-time users compared to cevimeline (i.e., 47% vs. 27%). Severe perspiration was the most frequent side effect leading to cessation of therapy and occurred more frequently among patients using pilocarpine (25%) compared to those treated with cevimeline (11%). This conclusion is in concurrence with the outcomes of the previously published study of Chainani-Wu et al. [76], according to which, sweating was reported more frequently with pilocarpine compared to bethanechol or cevimeline. Additionally, pilocarpine may interact with various medications, including beta adrenergic antagonists and other parasympathomimetic drugs, and could antagonize the therapeutic anticholinergic effects of medications, such as oxybutynin.

## 5. Topical Administration of Pilocarpine

Within this set framework, new strategies to prepare novel and effective dosage forms for topical (in the mouth) pilocarpine administration, in order to enhance salivary flow with minimal systemic manifestations, have emerged. Several studies have been performed, especially during the last decade, regarding the efficacy of pilocarpine topical delivery systems, such as mouthwashes, tablets, lozenges and mouth sprays [77]. According to the findings, which are to be presented in detail afterwards, the topical delivery of pilocarpine can be regarded as equivalent to systemic delivery of the drug, efficacy-wise, but with improved patient tolerance.

One of the earliest studies concerning the topical administration of pilocarpine was conducted by Rhodus et al., a single-blind, placebo-controlled study evaluating the efficacy of long-term pilocarpine solution administration topically [78]. Specifically, 18 patients with diagnosed Sjögren’s syndrome were divided into two groups: the pilocarpine-treated and the placebo group. A liquid drop preparation containing 2% pilocarpine was administrated to the patients of the first group for 6 weeks, with the dosage regimen being four drops three times per day. Simultaneously, the patients of the control group were given a placebo of deionized water with identical appearance, at the same dosage, for 6 weeks. According to the obtained results, a significant overall increase in both whole unstimulated saliva and parotid stimulated saliva was presented in patients treated with pilocarpine compared to the placebo group. Concerning the adverse side effects, these were negligible. Specifically, no participant at this study presented any type of cardiovascular or respiratory disease or changes in pulse rate, rhythm or blood pressure. 

A few years later, twenty patients with radiation-induced xerostomia were enrolled into a prospective randomized crossover trial comparing a mucin-based artificial saliva (Saliva Orthana, A.S Pharma, Faridabad, India) and a mouthwash containing pilocarpine. The specific trial performed by Davies et al. [79] is considered to be the first study comparing an artificial saliva with pilocarpine in the treatment of radiation-induced xerostomia. Each patient received initially a three-month treatment with the Saliva Orthana—the recommended dose was 2–3 sprays when required—and, subsequently, a three-month treatment with the pilocarpine mouthwash—he dosage regimen was 5 mg three times a day. There was a washout period between the two treatments lasting one week. Overall, mouthwash presented an increased effectiveness in relieving xerostomia’s symptoms compared to artificial saliva, a phenomenon being empirically confirmed by an impressive 47% of patients claiming their preference of continuing with this treatment after the end of study. 

Thereafter, a plethora of studies evaluating the performance of different pilocarpine mouthwashes was published. 

**Figure 4:**
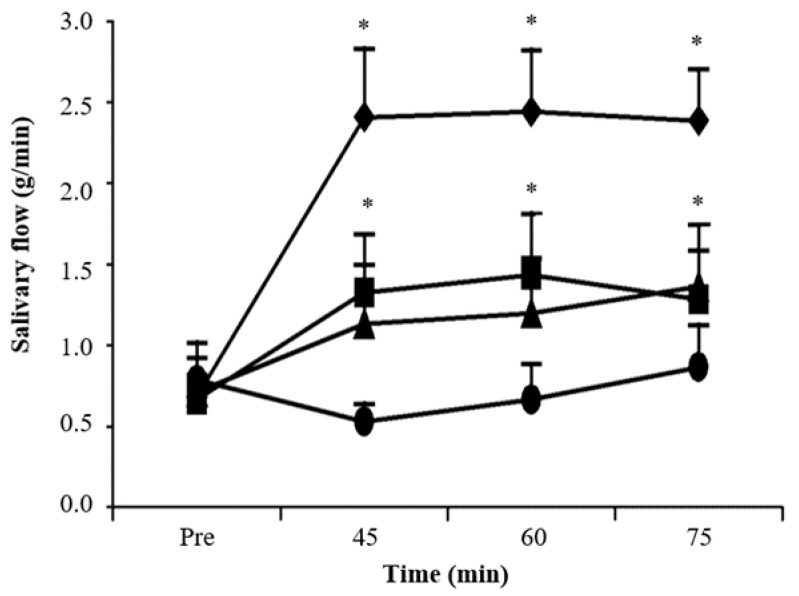
Salivary flow (g/min) prior (Pre) to pilocarpine treatment and 45, 60 and 75 min after mouth washing with 10 mL of 0.5 (triangles), 1 (squares) or 2% (rhombus) pilocarpine solutions or saline (cycles) as the control solution for 1 min. * *p* < 0.05 compared to control (Student-Newman-Keuls test). Adapted from [80].

Specifically, in a clinical trial prepared by Bernardi and co-workers [80], the impact of pilocarpine solutions, such as mouthwashes on salivary flow, as well as the appearance of any adverse effect on healthy participants, were both evaluated. Forty volunteers received 10 mL of 0.5, 1 and 2% pilocarpine solutions or 0.9% saline in a randomized, double-blind, placebo-controlled manner. Salivation was measured before, as well as 45, 60 and 75 min after mouth rinsing for 1 min with 10 mL of saline or pilocarpine solutions. As illustrated in Figure 4, a comparative analysis indicated the comparative advantage of mouth washing with 1 or 2% pilocarpine solution, as they had both increased the salivary flow compared to the saline solution, while salivation remained indifferent at 45, 60, 75 after mouth rinsing with pilocarpine solutions. In particular, mouth rinsing with 1% or 2% pilocarpine solutions induced a significant objective and subjective dose-dependent increase in salivary flow, similar to the effect of the orally administrated pilocarpine. Cardiovascular, visual, gastrointestinal and behavioral signs did not change after the topical administration of pilocarpine. Hence, in this study the use of pilocarpine mouthwash solutions resulted in increased salivary flow in healthy volunteers, presenting—quite importantly—zero adverse effect status.

In another study carried out by Kim and coworkers [81], the efficacy of a 0.1% pilocarpine mouthwash on patients with xerostomia was determined. Sixty volunteers were randomly allocated in two groups. The pilocarpine group, which was regarded as the experimental group, was treated with a 0.1% pilocarpine solution and the control group used 0.9% saline. Palatal and labial secretions were significantly higher in the experimental group than in the control group at 0, 30 and 60 min after mouth washing. Moreover, the unstimulated whole salivary flow rate was increased in the pilocarpine-treated group and differed from that in the control group. Interestingly, after 4 weeks, the severity of oral dryness was decreased in both groups with no difference between them. No adverse effects were reported on the questionnaire during the experimental time period in both groups.

In a follow up study, the efficacy and the safety of pilocarpine mouthwash in elderly patients with xerostomia was evaluated by Tanigawa et al. [82]. Forty elderly patients were randomly divided into a pilocarpine mouthwash or water rinse (control) group and the outcomes were assessed before and one month after treatment. An overall improvement was observed in 47% of the pilocarpine group compared to 14% of the control group, while the stimulated salivary flow rate was significantly increased after pilocarpine mouthwash treatment. As for the presented adverse side effects, five of the participated patients reported side effects after pilocarpine mouthwash use, predominantly limited to oral discomfort. Consequently, this study showed that pilocarpine mouthwash relieved dry mouth symptoms and improved saliva production with minor side effects in elderly patients.

Another landmark study at the pilocarpine topical administration field is the one carried out by Park et al. [83], since a direct comparison between the effects of a pilocarpine mouthwash on salivary flow and those of the systemic administration of 5 mg pilocarpine tablet in healthy subjects took place. The innovative element of this specific study is based on the direct comparison of the examined corresponding efficacies of the topical delivery systems and the orally administrated pilocarpine, instead of sticking to the common strategy followed by previous studies that compare placebo formulation or artificial saliva. Specifically, 12 healthy volunteers were enrolled at this double blind, placebo-controlled, crossover clinical trial. Each volunteer was administered 5 mg-pilocarpine tablet, 4 mL of 2% pilocarpine solution and placebo solution in a predetermined order with a wash-out period of at least two days between sessions. Blood pressure and pulse rate were also measured, and subjective effect and potential side effects were evaluated by a self-administrated questionnaire. The findings of this study demonstrated that the mouthwash containing 2% pilocarpine increased the salivary flow rate in healthy subjects compared to placebo and its effect was comparable to that of the 5 mg pilocarpine tablet. 

The promising results of the 2% pilocarpine solution having been proven, the effects of various mouthwashes with different concentrations of pilocarpine on healthy volunteers were afterwards examined in the study of Song et al. [84]. In this research, 30 healthy volunteers were randomly divided into 6 groups, each one being treated with a different concentration of pilocarpine mouthwash, namely 0.1%, 0.5%, 1.0%, 1.5%, 2.0% and the placebo. Even though the concentrations of 0.5% and 0.1% had no effects on salivation, the salivation of the higher concentration groups (i.e., pilocarpine concentration ≥ 1%) was significantly increased without any serious side effects. Similarly, a clinical, randomized, placebo-controlled trial conducted by Beatris and coworkers [85] with 36 healthy individuals proved the dose and time dependency of the increase in salivation. Salivation was measured before and 15, 30, 45, 60 and 75 min after the administration of pilocarpine solutions (1% or 2%) or saline solution control. The 2% pilocarpine solution showed a significant increase in the salivation level of the volunteers 60 and 75 min after the mouthwash, compared to those who received saline.

Notwithstanding the fact that the majority of topical pilocarpine delivery systems concern mouthwashes, as proven by the above-mentioned studies, highlighting published trials evaluating the performance of pilocarpine mouth sprays or lozenges is definitely something that should not be omitted. Characteristically, Hamlar et al. [86] evaluated the effectiveness of pilocarpine hydrochloride suspended in a candylike pastille as a topical treatment for radiation-induced xerostomia in head and neck cancer patients. Forty previously irradiated patients participated in a prospective, randomized, double-blind, placebo-controlled trial and received increasingly higher pilocarpine dosages in pastilles for 5 successive weeks. Despite the fact that no significant increase in salivation was noted at each successive dose of pilocarpine, 74% of the patients reported that pilocarpine alleviated their subjective xerostomia. Overall, the topical pilocarpine administration presented similar results to previous systemic delivery methods for radiation-induced xerostomia, but with improved patient tolerance. Later, the research team of Taweechaisupapong [59], in a double-blinded, placebo-controlled trial, studied the effect of pilocarpine lozenges in the clinical symptoms and the salivary function of patients suffering from radiation-induced xerostomia. Thirty-three patients were randomly assigned to receive Salagen^®^ tablet, pilocarpine hydrochloride lozenge (3 or 5 mg) or placebo lozenge every 10 days. At this point, it should be highlighted that this is the first study evaluating the effectiveness of the developed topical pilocarpine delivery system via a comparison with the market commercial formulation. According to the obtained results, patients treated with 5-mg pilocarpine lozenges showed the best clinical responses when both safety and efficacy were considered.

Mouth sprays constitute another category of topical delivery formulation examined for pilocarpine administration. Initially, Frydrych et al. [38] recruited 23 patients diagnosed with radiation-induced hyposalivation for a randomized, double-blind trial aiming to investigate the effectiveness of pilocarpine in relieving xerostomia symptoms when administered in a mouth spray. Patients were allocated in a random manner to either pilocarpine or placebo medicaments used for eight weeks. 

Both stimulated and unstimulated salivary flow rates appeared to be ameliorated in all patients with residual functioning salivary tissue having utilized pilocarpine, despite an early withdrawal of three—prior to the time defined as sufficient for conclusions—because of adverse side effects, such as diarrhea, constipation and increased dryness of the mouth. In addition, when the effectiveness of a pilocarpine spray as a treatment for xerostomia through a prospective, randomized, double-blind, crossover, placebo-controlled clinical trial was examined by the research team of Santos Polvora [87], the salivary flow of the pilocarpine-treated participants was significantly increased after the utilization of the spray. Yet, it is worth mentioning that these results are in disagreement with the findings of the study carried out by Pereira et al. [88], according to which the performance of the evaluated pilocarpine spray was similar to that of the placebo on the patient’s stimulated whole saliva flow. In this specific study, 40 patients suffering from radiation-induced xerostomia were randomly assigned to either the placebo or pilocarpine spray group.

**Figure 5:**
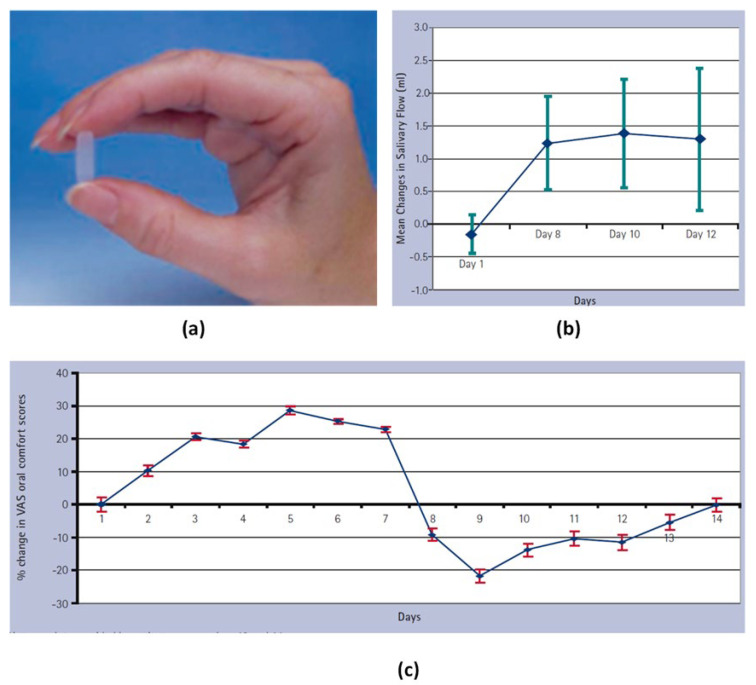
The hydrogel polymeric buccal formulation inserts (**a**), mean change in the salivary flow rates (**b**) and percentage change in visual analogue scale regarding oral comfort scores in patients (**c**). Adopted from [91].

Given the fact that mouthwashes or oral rinses target mainly the minor salivary glands, which produce 10% of the total salivary secretions [89,90], some novel pilocarpine formulations have been proposed in order to target the major salivary glands. For example, Gibson et al. [91] designed a novel hydrogel polymer buccal insert containing 5 mg pilocarpine and evaluated its effect on the patients’ saliva production and the referred tolerability (Figure 5a). The new formulation was designed for releasing the drug in a controlled fashion over a three-hour period. Specifically, 8 patients with Sjögren’s syndrome participated in an open, uncontrolled pilot study for 14 days. The obtained results showed that the salivary and lacrimal secretions in the majority of patients were increased (Figure 5b), while there was a noticeable improvement in oral comfort scores after the first administration of the pilocarpine inserts (Figure 5c). Additionally, the inserts were well tolerated by all patients, since the referred adverse effects were limited and none of them was characterized as a serious one.

**Figure 6:**
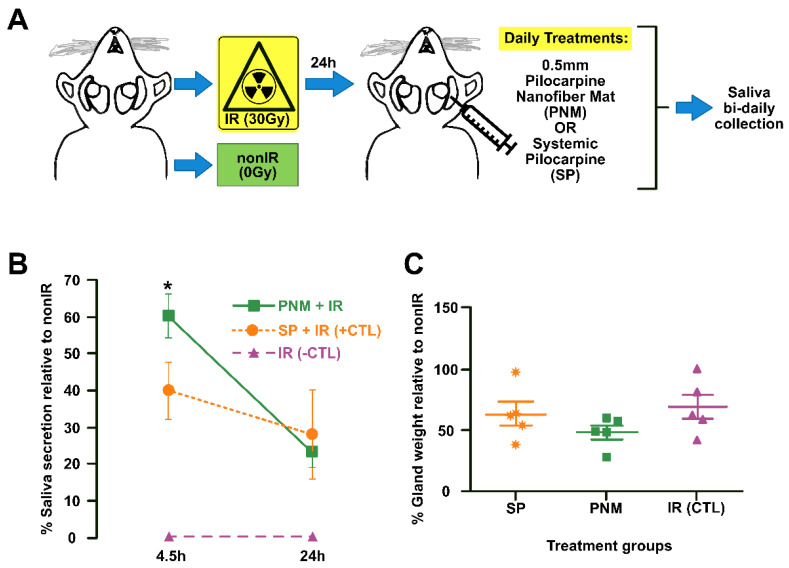
Schematic drawing of the in vivo salivary glands hypofunction model (**A**), one day saliva secretion rate after administration of the pilocarpine nanofiber mats (PNM) and the orally administrated systemic pilocarpine (SP) (**B**) and salivary gland weight remained unchanged PNM administration (**C**). Error bars represent SEM from *n* = 4−5. * *p* < 0.05 when compared to irradiated group with systemic pilocarpine only (SP, which represents the positive CTL). IR: irradiated (negative CTL). nonIR: non-irradiated control group. SGs: salivary glands. Adapted from [92].

In a more recent study, Muthumariappan and coworkers [92], in order to overcome the side effects induced by the systematic oral administration of pilocarpine, evaluated the use of a novel localized formulation of pilocarpine targeting the salivary glands. The proposed formulation consisted of pilocarpine-loaded Poly(lactic-co-glycolic acid) (PLGA)/poly(ethylene glycol) (PEG) nanofiber mats via an electrospinning technique. Drug release studies presented an initial burst release of one-fourth of the encapsulated pilocarpine after 4.5 h, which was subsequently flattened, obtaining over time a release profile much more gradual and steadier. Finally, the authors studied in vivo the efficacy of the prepared pilocarpine mats compared to conventional oral administration in a mouse acute hypofunctional salivary glands model (Figure 6A). The result showed that 4.5 h the new topical formulation significantly increased the saliva secretion, although after one day, no differences were recorded between the two tests (Figure 6B,C). Additionally, the no gross changes in salivary glands composition and cellular content were recorded between the two tested regimes. Overall, this study highlights that an intradermal pilocarpine loaded PLGA/PEG nanofiber mat is a promising system for potential clinical use as an intradermal formulation for the early and prompt treatment of xerostomia.

**Figure 7:**
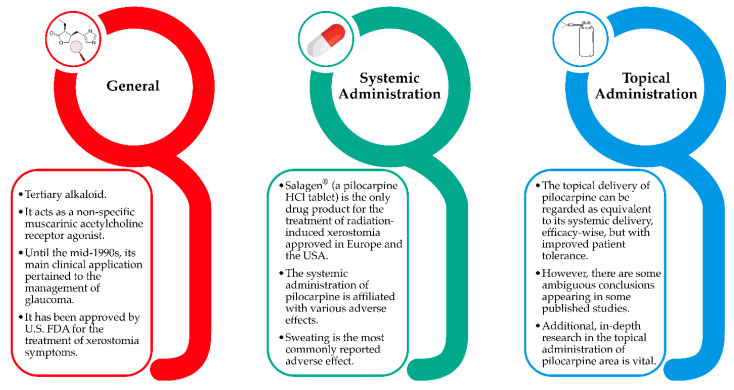
The most important points of pilocarpine’s administration.

To sum up, the most important points of pilocarpine’s administration are presented in Figure 7, while the above-mentioned studies are presented in chronological order in Table 2. As it can be easily concluded, their significant results, as well as some ambiguous conclusions or inconsistencies appearing in some of them, render the additional in-depth research on the topical administration of pilocarpine area vital.

## 6. Conclusions

Generally, patients with xerostomia suffer symptoms that significantly affect their life quality, since xerostomia is responsible for an increased incidence of dental caries, oral fungal infections (e.g., candidiasis) and periodontal disease. Pilocarpine is the most commonly used medication and has also been approved by U.S. FDA for the treatment of Sjögren’s syndrome and the relief of radiation-induced xerostomia’ symptoms. Notwithstanding the fact that there is a plethora of published studies proving the effectiveness of pilocarpine’s oral administration against xerostomia, the referred adverse side effects of pilocarpine pose a challenge that needs to be surpassed during the development of a management approach. In this vein, there is an urgent need to prepare novel and effective formulations for topical pilocarpine administration in order to enhance salivary flow with minimal systemic manifestations. Several studies have been performed, especially during the last decade, regarding the efficacy of pilocarpine topical delivery systems. According to the findings, the topical delivery of pilocarpine can be regarded as equivalent to systemic delivery of the drug associated, however with significantly less adverse effects. However, the significant results of these studies, as well as some inconsistencies amongst them, render the additional in-depth analysis of pilocarpine topical administration not only crucial from a scientific perspective, but also promising and useful commercially. 

## Figures and Tables

**Table 1:** Randomized, double-blind, placebo-controlled studies evaluating the clinical efficacy of oral pilocarpine in improving salivary gland flow and relieving symptoms of xerostomia caused by either radiation therapy of the head and neck or salivary gland dysfunction.

Reference	Year of Publishment	Number of Patients	Diagnosis	Administrated Dose of Pilocarpine and Duration	Outcome/Results
Fox et al. [42]	1986	6	Inflammatory exocrinopathy.	5.0 mg once a day (o.d) for 2 days.	Reduction in oral dryness and increased salivation in all patients.
Creenspan et al. [43]	1987	12	Radiation-induced xerostomia.	5.0 or 7.5 mg three times a day (t.i.d) or four times a day (q.i.d) for 90 days.	75% of the patients treated with pilocarpine experienced significant improvement of the mean stimulated whole salivary and parotid salivary flow rate.
Schuller et al. [44]	1989	14	Radiation-induced xerostomia.	3.0 mg t.i.d	60% of the patients treated with pilocarpine presented increased whole salivary flow rate after 6 weeks.
Fox et al. [45]	1991	39	Sjögren’s syndrome (21 patients), radiation-induced xerostomia (12 patients), idiopathic salivary gland dysfunction (6 patients).	5.0 mg t.i.d for 5 months.	~68% of the patients treated with pilocarpine (i.e., 21/31) presented significantly increased salivary flow. Moreover, 27/31 participants reported a subjective amelioration in oral dryness feeling, as well as in the processes of speaking, chewing and swallowing.
LeVeque et al. [46]	1993	156	Radiation-induced xerostomia.	2.5 mg t.i.d increased up to 10 mg t.i.d for 12 weeks.	Pilocarpine significantly ameliorated overall global assessments as compared to placebo.
Valdez et al. [47]	1993	9	Radiation-induced xerostomia.	5.0 mg q.i.d for 3 months.	A lower frequency of oral symptoms during treatment was reported in the pilocarpine-treated group, as compared to the placebo-treated group.
Johnson et al. [48]	1993	207	Radiation-induced xerostomia.	5.0 mg or 10.0 mg tablets t.i.d for 12 weeks.	44 and 46% of patients treated with 5 and 10 mg, respectively, claimed that their feeling of oral dryness was improved. Moreover, 31 and 37% of patients treated with 5 and 10 mg, respectively, referred improved mouth and tongue comfort.
Rieke et al. [49]	1995	369	Radiation-induced xerostomia.	5.0 mg or 10.0 mg tablets t.i.d for 12 weeks.	Statistically significant improvements in salivary flow in pilocarpine treatment groups.
Zimmerman et al. [50]	1997	44	Radiation-induced xerostomia.	5.0 mg q.i.d for 18 months.	Oral pilocarpine usage during and 3 months thereafter radiotherapy, appeared to have contributed to a significantly less subjective xerostomia, as compared to patients excluded from pilocarpine treatment.
Vivino et al. [51]	1999	373	Primary or secondary Sjögren’s syndrome.	2.5 mg or 5.0 mg q.i.d for 12 weeks.	Patients administrated with 5 mg pilocarpine 4 times daily reported a decent tolerance, as well as an important improvement in dry mouth symptoms.
Horiot et al. [52]	2000	145	Radiation-induced xerostomia.	5.0 mg t.i.d for 12 weeks.	97 patients (~67%) mentioned a significant alleviation of xerostomia’s symptoms at 12 weeks. 38 patients (26%) stopped treatment because of acute intolerance (sweating, nausea, vomiting) or no response.
Mateos et al. [53]	2001	49	Radiation-induced xerostomia.	5.0 mg t.i.d (started the day before radiation treatment and continued throughout the first year of follow-up).	The differencies between patients treated with pilocarpine and those receiving placebo was not statistically significant.
Haddad et al. [54]	2002	39	Radiation-induced xerostomia.	5.0 mg t.i.d (started with radiation and continued until 3 months after the end of radiotherapy.	As compared to placebo, pilocarpine administrated at radiotherapy was capable of resulting in a remarkable alleviation of xerostomia.
Warde et al. [55]	2002	130	Radiation-induced xerostomia.	5.0 mg t.i.d (starting from day 1 of the radiation and continuing for 1 month after the end of the treatment).	No therapeutic effect of pilocarpine on radiation-induced xerostomia was pointed out.
Fisher et al. [56] (ClinicalTrials.gov ID: NCT00003139)	2003	213	Radiation-induced xerostomia.	5.0 mg q.i.d (starting before the radiation treatment until 3 or 6 months after treatment).	Concomitant use of pilocarpine maintained and protected unstimulated salivary flow.
Gornitsky et al. [57]	2004	58	Radiation-induced xerostomia.	1st study phase: 5.0 mg five times a day (from the first to the last day of radiation treatment) 2nd study phase (post-radiation): 5.0 mg q.i.d.	Pilocarpine reduced discomfort and pain symptoms as well. An improved global quality of life was reported only at the conclusion of the first study phase.
Papas et al. [58] (ClinicalTrials.gov ID: NCT04470479)	2004	256	Sjögren’s-related xerostomia.	5.0 mg q.i.d for six weeks and then 7.5 mg q.i.d for the next 6 weeks.	A remarkable relief in dry mouth symptoms was noted at 20 mg/day.
Taweechaisupapong et al. [59]	2006	33	Radiation-induced xerostomia.	3.0 or 5.0 mg every ten days.	There was statistically significant increase in salivary production in pilocarpine treatment groups vs. placebo.
Scarantino et al.[60] (ClinicalTrials.gov ID: NCT00003139)	2006	245	Radiation-induced xerostomia.	5.0 mg q.i.d.	The overall results for salivary function at 3 and 6 months demonstrated statistically significant differences in favor of the pilocarpine arm for unstimulated salivary flow.
Wu et al. [61]	2006	44	Sjögren’s syndrome.	5.0 mg q.i.d for 12 weeks.	Pilocarpine treatment managed a significant amelioration of mouth dryness-related symptoms and saliva production, as compared to placebo.
Chitapanarux et al. [62]	2008	33	Radiation-induced xerostomia.	5.0 mg t.i.d (starting from the 1st of the radiation and continuing for 3 month after the end of the treatment).	Improvement of xerostomia symptoms was observed, with a mean total subjective xerostomia score improvement at the first 4 weeks of oral pilocarpine treatment.
Cifuentes et al. [23] (ClinicalTrials.gov ID: NCT04470479)	2018	72	Sjögren’s syndrome.	5.0 mg t.i.d for 12 weeks.	Patients treated with pilocarpine showed a statistically significant improvement in their salivary flow, lachrymal flow, as well as their subjective global assessment, as compared to the patients administrated artificial saliva

**Table 2:** Published studies evaluating the clinical efficacy of topical pilocarpine delivery formulations in improving salivary gland flow and relieving symptoms of xerostomia. The studies are presented in chronological order.

Reference	Year of Publishment	Volunteers	Drug Formulation for Topical Administration	Reference/Outcome
Rhodus et al. [78]	1992	18	Pilocarpine ophthalmic solution	Both whole unstimulated salivary flow and parotid stimulated salivary flow presented a significant improvement in the pilocarpine group, compared to those of the placebo group.
Davies et al. [79]	1994	20	Mouthwash	The increased pilocarpine mouthwash effectiveness, compared to that of the artificial saliva in relieving the patients’ symptoms was noted by patients.
Hamlar et al. [86]	1996	40	Candy-like pastilles	The alleviation of subjective xerostomia’s symptoms was reported by 74% of patients. Moreover, the topical pilocarpine administration approached the same level of efficacy compared to previous delivery methods for radiation-induced xerostomia yet presenting the comparative advantage of a significantly improved patient tolerance.
Bernardi et al. [80]	2002	40	Mouthwash	The results of pilocarpine mouthwash solutions in increasing salivary flow in healthy participants was proved, with no adverse effects.
Frydrych et al. [38]	2002	23	Mouth spray	All patients treated with pilocarpine demonstrated improvement in stimulated and unstimulated salivary flow rates. Candida counts decreased among the cases.
Taweechaisupapong et al. [59]	2006	33	Lozenges	Salivary production in pilocarpine treatment group, as compared to that of the placebo group, appeared a statistically significant improvement. The 5 mg pilocarpine lozenge claims the top spot, as far as the clinical results are concerned.
Gibson et al. [91]	2007	8	Hydrogel buccal inserts	Better oral and ocular scores, along with a generally ameliorated saliva production were noted, while all patients, with the exception of one who wore dentures, agreed on the decent tolerance of the inserts.
Kim et al. [81]	2014	60	Mouthwash	The unstimulated whole salivary flow rate was increased.
Tanigawa et al. [82]	2015	40	Mouthwash	47% of patients treated with pilocarpine reported an overall improvement. Moreover, following pilocarpine mouthwash treatment, the stimulated salivary flow rate appeared to be significantly increased, along with a predominant attenuation of side effects referred after pilocarpine mouthwash use to oral discomfort.
Park et al. [83]	2015	12	Mouthwash	The examined 2% pilocarpine solution as mouthwash increased salivary flow rate, compared to the placebo solution. Its efficacy was comparable to pilocarpine tablet, yet with the comparative advantage of presenting reduced side effects in healthy subjects.
Song et al. [84]	2017	30	Mouthwash	Pilocarpine mouthwash with at least 1.0% concentration and at a more-than- a-minute application might be clinically effective without any serious side effects.
Beatris et al. [85]	2018	36	Mouthwash	Treatment with pilocarpine mouthwash provided an increased salivation, without being followed any significant clinical adverse effect.
Watanabe et al.[93]	2018	24	Mouthwash	This new, low-dose pilocarpine formulation was well-tolerated and resulted to significant improvements in dry mouth symptoms and other xerostomic conditions in patients with Sjögren’s syndrome.
Santos Polvora et al. [87]	2018	28	Mouth spray	The pilocarpine spray significantly increased the salivary flow and alleviated xerostomia symptoms.
Pereira et al. [88]	2020	40	Mouth spray	The pilocarpine spray presented no significant differences as compared to placebo.
Sarideechaigul et al. [94]	2021	31	Artificial saliva	The evaluated formulations with were regarded as safe with minimum referred adverse effects. Specifically, while some adverse effects were in fact mentioned, they were not regarded as severe.
Gusmão et al. [95]	2021	25	Mucoadhesive tablets	The mucoadhesive tablets resulted to higher salivary concentrations of pilocarpine as compared to the conventional oral tablet.

## Data Availability

No new data were created or analyzed in this study. Data sharing is not applicable to this article.
